# The Metric System in Medicine

**Published:** 1880-12

**Authors:** 


THE METRIC SYSTEM IN MEDICINE.

THE late meeting of the American Medical Association recommended the universal adoption of the metric system by physicians, and of course by pharmacists. It will be no easy task to carry out the resolve. Such a revolution as it would involve was never accomplished since the foundation of human society unless by slow degrees. Dr. Gaillard, in his *Medical Journal*, speaks out on the subject, thus:

"The adoption of the report on the metric system and the recommendation for the general adoption of the metric system in this country was, to say the least, Quixotic. The adoption of this system by American physicians is impracticable and the Association only emasculates itself by lending its influence in behalf of measures which however good in themselves are absolutely impracticable. Its action will be absolutely without result, and the powerlessness of the association will be lamentably and uselessly manifested. If the metric system could be universally enforced by law in this country, such a result would be a blessing, but apart from such a law it is more than idle to expect that one of the departments of business in America should adopt such a system, while in every other avocation it is repudiated.—*Pacific Medical and Surgical Journal.*

[We republish the above as a most remarkable plea. That the prophesy is untrue, has already been proved by the successful adoption of the metric system by the Medical Club of this city.—ED.]

